# Immune checkpoint inhibitors against thyroid cancer

**DOI:** 10.1097/JS9.0000000000002830

**Published:** 2025-07-02

**Authors:** Kun Zhao, Libo Chen

**Affiliations:** a Department of Nuclear Medicine, Shanghai Jiao Tong University Affiliated Sixth People’s Hospital South Campus, Shanghai, China; b Department of Nuclear Medicine, Shanghai Sixth People’s Hospital Affiliated to Shanghai Jiao Tong University School of Medicine, Shanghai, China

**Keywords:** clinical trials, immune checkpoint inhibitors, therapeutic strategies, thyroid cancer

## Abstract

**Purpose:**

This study comprehensively analyzed the global clinical trials and depicted a landscape of immune checkpoint inhibitors (ICIs) in thyroid cancer therapy.

**Methods:**

This study leveraged the Trialtrove database (https://clinicalintelligence.citeline.com/) to conduct a systematic analysis of the clinical trial landscape in thyroid cancer research. Utilizing professional medical subject headings, we performed a comprehensive search with keywords such as “Immune checkpoint inhibitor” and “Thyroid oncology,” identifying a total of 113 clinical trials.

**Results:**

Our review of 113 trials revealed a peak in 2020, primarily driven by phase II and III studies. Pembrolizumab was the most frequently studied monotherapy, while combinations such as Pembrolizumab-Lenvatinib and Camrelizumab-Apatinib were also evaluated. Biomarkers like BRAF and immune checkpoint markers were prominently investigated. Although most trials achieved their primary endpoints, only five reported positive outcomes. Our analysis underscores the importance of specific thyroid cancer subtypes and novel combination therapies in advancing ICIs applications.

**Conclusion:**

The study presents some valuable insights regarding the present state and future perspectives of clinical trials on thyroid cancer

Immune checkpoint inhibitors (ICIs) have demonstrated efficacy in cancer treatment, with durable responses and enhanced overall survival observed both as monotherapy and in combination with chemotherapy or other immunotherapies^[1,2]^. However, their application in thyroid cancer has been impeded by the lack of robust endpoints to evaluate efficacy, particularly in neoadjuvant settings where rapid and reproducible clinical activity endpoints are essential. Several trials have investigated ICIs used alone or in conjunction with chemotherapy across different treatment phases, including adjuvant, neoadjuvant, and perioperative settings in thyroid cancer^[3,4]^. We discuss the role of ICIs in thyroid cancer treatment, evaluates current clinical strategies, and discusses the evolving trial landscape. Furthermore, we propose approaches to maximize their therapeutic potential in future clinical practice and ensure that our study is compliant with the Transparency In The reporting of Artificial INtelligence (TITAN) Guidelines 2025^[5]^.

**HIGHLIGHTS**

- A total of 113 global clinical trials using ICIs for thyroid cancer were analyzed using data from Trialtrove database.
- Pembrolizumab is the most frequently studied monotherapy, while Pembrolizumab -Lenvatinib and Camrelizumab-Apatinib are notable combination therapies under evaluation, reflecting their potential in thyroid cancer treatment.
- Biomarkers such as BRAF and immune checkpoint markers are prominently investigated, with the BRAF V600E variant being crucial for treatment stratification, highlighting the importance of understanding the disease’s molecular characteristics.
- While most trials achieved their primary endpoints, only five reported positive outcomes in terms of overall efficacy, with key drugs like Pembrolizumab, Lenvatinib, Camrelizumab, Apatinib, Durvalumab, and Atezolizumab showing promise. This highlights the ongoing challenges in achieving overall efficacy with ICIs in thyroid cancer.
- Our study emphasizes the need for further investigation into combination therapies, long-term safety data, and resistance mechanisms in order to enhance the efficacy and durability of ICIs treatments for thyroid cancer.

This study leverages the Trialtrove database (https://clinicalintelligence.citeline.com/) to conduct a systematic analysis of the clinical trial landscape in thyroid cancer research. Utilizing professional medical subject headings, we performed a comprehensive search with keywords such as “Immune checkpoint inhibitor” and “Thyroid oncology,” identifying a total of 113 clinical trials. The research further explores different phases of clinical trials, the types of drugs employed, and offers a detailed overview of the latest advancements and future directions in thyroid cancer treatment.

We systematically reviewed data from 113 clinical trials (Supplemental Digital Content, Table, available at: http://links.lww.com/JS9/E587). The annual distribution of these trials exhibited a peak in 2020, primarily driven by an increase in phase II and III studies, followed by a sustained downward trend from 2020 to 2024 (Fig. 1A). To date, 41 trials have been completed, while 39 are currently open. Other trials are at various stages, including closed, temporarily closed, planned, or terminated (Fig. 1B). Pembrolizumab was most frequently studied as a monotherapy, with a total of 10 trials. Additionally, the Pembrolizumab-Lenvatinib combination was assessed in a total of 5 trials. Furthermore, the coupling of camrelizumab and apatinib was involved in 4 trials (Fig. 1C). The bar chart illustrates the frequency of biomarkers examined in thyroid cancer clinical research. BRAF is the most frequently studied biomarker, reflecting its pivotal role in disease progression and targeted therapy development. GPT ranks second; however, its specific relevance necessitates further mechanistic investigation (Fig. 1D). The BRAF^V600E^ variant was prominently present, underscoring its importance as a molecular subtype in treatment stratification. Immune checkpoint markers PDCD1 and CD274 were also featured prominently, noting the expanding role of immunotherapy in treatment strategies. Additionally, ERBB2, CD8A, and CD4 reflect two research priorities: (1) modulation of the immune microenvironment (e.g., CD8+/CD4+ T-cell infiltration) and (2) research on oncogenic signaling pathways (e.g., ERBB2). While most trials met their primary endpoints, only five reported positive results, involving key drugs such as Pembrolizumab, Lenvatinib, Camrelizumab, Apatinib, Durvalumab, and Atezolizumab (Table 1).

**Figure 1.**
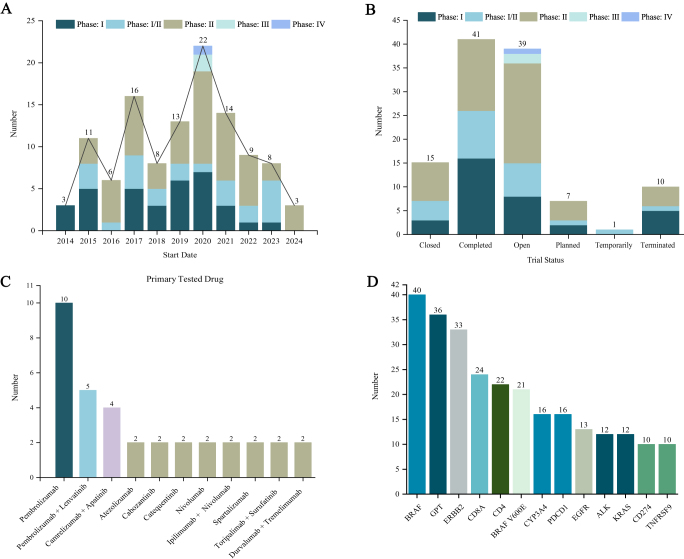
Clinical landscape of ICIs in the treatment of thyroid cancer. A. Distribution and status of thyroid cancer clinical trials across different phases from 2014 to 2024. B. Distribution of thyroid cancer clinical trials by status and phase (I–IV). C. Distribution of various drug types in thyroid cancer clinical trials. D. Frequency of biomarkers examined in thyroid cancer clinical trials.

**Table 1:** Summary of completed clinical trials of ICIs for thyroid cancer that had positive outcomes or met primary endpoints

ID	Title	Phase	Patient segment	Drug	outcome details
537250	Camrelizumab plus apatinib mesylate in patients with recurrent or metastatic poorly differentiated thyroid carcinoma (PDTC) /anaplastic thyroid carcinoma (ATC): A prospective, open-label, phase II study	II	Anaplastic; BRAF; Line of therapy N/A; Metastatic; Papillary; PD-1 Naive; PD-L1 Naive; PD-L1 Positive	Apatinib Camrelizumab	The objective response rate was 53.3%, and the disease control rate was 60%.Camrelizumab combined with apatinib mesylate had promising antitumor activity and manageable toxicities in recurrent or metastatic PDTC/ATC.
354199	Phase II, Open-label, Study in Patients with Anaplastic (ATC) or Poorly Differentiated Thyroid Carcinomas (PDTC) to Investigate the Clinical Efficacy and Safety of the Combination Therapy of Lenvatinib and Pembrolizumab.	II	Anaplastic; Follicular; Second line	Lenvatinib Pembrolizumab	The primary endpoint ORR at 3 mo was achieved. Our results implicate that a combination of lenvatinib and pembrolizumab is safe and effective in patients with ATC and PDTC, and induces high response rates including long-lasting remissions.
305133	Radioiodine (RAI) in Combination With Durvalumab (Medi4736) for RAI-avid, Recurrent/Metastatic Thyroid Cancers	I	Follicular; Fourth line or greater; Metastatic; Papillary; PD-1 Naive; PD-L1 Naive; Recurrent	Durvalumab	No DLTs or >Grade 3 durva related adverse events (AEs) were observed. Durva plus RAI is safe and well tolerated. The preliminary efficacy signal in this small cohort is promising.
303142	Atezolizumab Combinations With Chemotherapy for Anaplastic and Poorly Differentiated Thyroid Carcinomas	II	Adjuvant; Anaplastic; BRAF; First line; Follicular; Metastatic; PD-1 Naive; PD-L1 Naive; PD-L1 Positive; Second line; Unresectable	Atezolizumab	Atezolizumab + vemurafenib/cobimetinib for BRAF-mutated or + cobimetinib for NF1/2 or RAS-mutated ATC is effective, as evidenced by the long OS in these pts (13 mos > historical control). A significant number of patients, particularly in cohort 1, were able to undergo complete tumor resection due to a favorable response to tx.
291402	Combination Targeted Therapy With Pembrolizumab and Lenvatinib in Progressive, adioiodine-Refractory Differentiated Thyroid Cancers: A Phase II Study	II	Anaplastic; Follicular; Medullary; Metastatic; Papillary; PD-1 Naive; PD-L1 Naive; Recurrent; Second line; Unresectable	Lenvatinib Pembrolizumab	Adding pembrolizumab to lenvatinib therapy is well tolerated in patients with RAIR DTC. A preliminary primary endpoint of ORR was 15%.

BRAF, B-Raf proto-oncogene serine/threonine kinase; DLTs, Dose- limiting toxicities; NF1/2, Neurofibromatosis type 1/2; ORR, Objective response rate; Programmed cell death ligand 1; PD-1, Programmed cell death protein 1; PD-L1, RAIR, Radioactive iodine-refractory; RAS-mutated, Rat sarcoma-mutated.

Over the past decade, the landscape of ICIs in thyroid cancer clinical trials has undergone a significant transformation. In 2020, the number of trials peaked, predominantly consisting of Phase II and III studies. This surge reflected the extensive exploration and validation of ICIs in thyroid cancer treatment to clarify their potential role in addressing this complex disease. However, following 2020, the number of trials declined. This was not a sign of waning research interest in ICIs, but rather a strategic shift in the research paradigm. The research community gradually moved away from broad and general trials towards more targeted and precise research methods. These methods primarily focused on specific subtypes of thyroid cancer, such as anaplastic thyroid cancer, which is aggressive and has limited treatment options, as well as advanced or metastatic differentiated thyroid cancer that is resistant to conventional therapies. Delving into these specific subtypes helps better understand their unique biological characteristics and immune microenvironments, and maximizing the clinical efficacy of ICIs.

In addition to the focus on specific subtypes, the shift in research is also prominently manifested in the exploration of novel combination therapies. Combining ICIs with other therapeutic approaches such as targeted therapy, chemotherapy, or radiotherapy has emerged as a promising research direction. For instance, the combination of ICIs with tyrosine kinase inhibitors (TKIs), which are commonly used in thyroid cancer treatment, is expected to enhance antitumor activity by targeting complementary pathways involved in tumor growth and immune evasion^[6,7]^. Similarly, the combination of ICIs and chemoradiotherapy may produce a synergistic effect^[8]^. These combination therapies aim to overcome resistance mechanisms, improve treatment outcomes, and expand the applications of ICIs in thyroid cancer treatment.

The frequent inclusion of Lenvatinib and Pembrolizumab in clinical trials evidences their established role in the treatment landscape, supported by their proven efficacy and favorable safety profiles. However, the participation of other agents, such as Apatinib, Camrelizumab, Durvalumab, and Atezolizumab, in multiple trials reflects ongoing efforts to develop additional therapeutic options. Although currently available data on these agents remain limited, preliminary evidence suggests they may produce synergistic effects when combined with other therapeutic modalities. For instance, Maria Cabanillas et al. demonstrated in their study that the combination of atezolizumab and targeted therapy significantly extended the median overall survival in patients with anaplastic thyroid carcinoma^[9]^.This underscores the necessity for further research into combination therapies to enhance treatment efficacy and optimize patient outcomes. Additionally, long-term safety data are also necessary to ensure the benefits of ICIs outweigh potential side effects, especially in combination therapies^[10]^, and ultimately enhance patient survival rates and quality of life.

The rapidly evolving landscape of ICI trials in thyroid cancer highlights the importance of targeted and combination approaches in enhancing treatment efficacy and optimizing patient outcomes. A deeper understanding of resistance mechanisms will be crucial for developing more effective and durable therapeutic strategies.

## Data Availability

The datasets generated and analyzed during the current study are available in the Trialtrove database (https://citeline.informa.com/trials/results).
